# Dental age estimation in southern Chinese population using panoramic radiographs: validation of three population specific reference datasets

**DOI:** 10.1186/s12880-018-0250-z

**Published:** 2018-04-27

**Authors:** Jayakumar Jayaraman, Graham J. Roberts, Hai Ming Wong, Nigel M. King

**Affiliations:** 1 School of Dentistry, International Medical University, Kuala Lumpur, Malaysia; 2 Department of Orthodontics, King’s College London Dental Institute, London, UK; 3 Paediatric Dentistry & Orthodontics, Faculty of Dentistry, Prince Philip Dental Hospital, The University of Hong Kong, 2/F, 34 Hospital Road, Sai Ying Pun, Hong Kong; 4 Paediatric Dentistry, School of Dentistry, University of Western Australia, Perth, Australia

**Keywords:** Panoramic radiographs, Age determination by teeth, Dental maturity, Southern Chinese

## Abstract

**Background:**

The accuracy of estimated age should depend on the reference data sets (RDS) from which the maturity scores or Ages of Attainment (AoA) were obtained. This study aimed to test the accuracy of age estimation from three different population specific dental reference datasets (RDS).

**Methods:**

Two hundred and sixty six dental panoramic radiographs of subjects belonging to southern Chinese ethnicity were scored and dental age (DA) was estimated from three reference datasets: French-Canadian, United Kingdom (UK) Caucasian and southern Chinese. Statistical significance was set at *p* < 0.05 and for each method, the difference between the chronological age (CA) and dental age (CA-DA) was calculated using paired t-tests. In addition, Chi-square tests were performed to evaluate the accuracy of the age estimates within specific time interval from CA.

**Results:**

The estimated age difference (CA-DA) using the French Canadian RDS was − 0.62 years for males and − 0.36 years for females. For the UK Caucasian RDS, the age difference was 0.25 years for males and 0.23 years for females. The difference observed using the southern Chinese RDS was − 0.02 years for both genders and the difference was not statistically significant (*p* > 0.05). The southern Chinese RDS estimated the age of 80% of subjects within ±12 months range, and 90% of subjects within ±18 months range (*p* < 0.05) showing it to be more accurate than other datasets.

**Conclusion:**

It is concluded that population specific Reference Data Sets improve the accuracy of dental age estimation.

## Background

Dental age estimation (DAE) is an important procedure in clinical science, forensic science, and legal proceedings. In clinical dentistry, it is used to determine the appropriate time to plan or implement treatment whilst in forensic investigations it assists in the identification of disaster victims [1]. Age estimation is extremely important when assessing the status of asylum seekers, especially when an individual’s age is disputed following loss of travel documents or false age claims. The process of the age assessment differs among countries and has been further modified by the legislation of the country [2]. Age can be estimated from different indicators that include physiological development, and skeletal and dental maturity. Dental age estimation is widely regarded as the method of choice as it has been proven to be both highly accurate and reliable [3]. However, there are few data available to determine the most appropriate method of age assessment to be employed in a particular country [4].

A panoramic radiograph serves as an excellent tool to visualize the entire dentition in a single image. The procedure for age assessment using radiographs involves two distinct phases; firstly, staging of the dental development, and secondly, retrieval of the scores or Ages of Attainment (AoA) for the corresponding stage of dental development from appropriate reference datasets. For this purpose, multiple methods for staging dental development have been proposed in the literature [5, 6]. The staging system developed by Demirjian and his co-workers classified dental development into 8 stages which is accepted as being the most reliable in terms of inter- and intra-examiner reliabilities [7]. The reference dataset serves as a standard from which the scores are obtained for age assessment calculation. A reference dataset has to be subjected to validation before its practical application. A landmark study on the construction of reference dataset was developed from the maturation of seven permanent left mandibular teeth of French-Canadian children. The development of teeth is shown to be bilaterally symmetrical so teeth present in the left side alone are included in the evaluation procedure. Subsequent to its development, this standard reference dataset for age estimations has been tested on various population groups. The accuracy of the age estimated from this dataset has been debated over many years; frequently it resulted in over estimation of the true chronological age. Furthermore, this particular method of age assessment has several other limitations, such as multiple calculations leading to potential errors, and inapplicability when there are bilaterally missing mandibular teeth. In addition, age estimations for subjects over 16 years of age are difficult because all of the teeth have completed their root formation [6]. Consequently, a reference data was recently created based on the maturity of both maxillary and mandibular teeth in a cohort of Caucasian children living in the United Kingdom (UK). This method has now superseded the Demirjian system and has been reported to be able to accurately estimate the age of Caucasian children [8].

Dental age estimation studies have been conducted on southern Chinese subjects. The applicability of two standard reference datasets, the French Canadian and the United Kingdom Caucasian were tested. These studies concluded that neither of these methods was suitable for age assessment of southern Chinese children due to differences between the chronological and dental ages in most age ranges. The chronological age of southern Chinese subjects was overestimated by the Demirjian French Canadian dataset and underestimated by the UK Caucasian reference dataset [9, 10]. These findings strongly supported the establishment of population specific dental reference dataset (RDS). Subsequently, a reference dataset has been developed based on the dental development of southern Chinese children and young adults. Furthermore, this has also been subjected to blind validation and has proved to be a reliable and accurate dataset for the age assessment for southern Chinese subjects [11]. Whilst genetics plays a greater role in the maturation of teeth, the influence of other factors including environment, nutrition and secular trends cannot be totally ignored during the selection of dataset suitable for age estimation. To add to this, there is controversy in the literature on the existence of population similarities and differences in dental maturation. A study reported similarities in dental maturation between Caucasian and Bangladeshi ethnic groups living in London [12]. This trend was also observed in the maturation pattern of permanent teeth in children in West Africa, the Middle-East and Europe.^13^ Several other studies acknowledged the presence of population differences and emphasized the need for ethnic specific standards for accurate age assessments [8, 11]. This raises a pertinent question; “how accurate are population specific datasets in dental age estimation?” This study aimed to answer this question by evaluating the accuracy of age estimation in southern Chinese subjects using three population specific reference datasets; (i) French Canadian, (ii) UK Caucasian, and (iii) southern Chinese. In addition, comparison of the dental age estimates within a specific time interval from chronological age could provide a better understanding on the accuracy of population specific datasets.

### Ethics, consent and permissions

Ethical approval for this study was obtained from the Institutional Review Board of the University of Hong Kong - West cluster Hospital authority (IRB No. UW 12–280). Written consent to participate in this study was obtained before the commencement of the study from every participant or parent (if the participant was under 18 years old).

## Methods

The study sample comprised of 266 healthy southern Chinese children and emerging adults with ages ranging from 2 to 21 years. The Dental Panoramic Tomograph (DPT) belonging to these subjects were randomly selected from the archives of the Prince Philip Dental Hospital, Hong Kong (GE 1000, Panelipse X-Ray Machine, General Electric Company, USA). A total of 14 subjects were included in each age range (2 to 3, 3 to 4, and up to 20 to 21 years; 19 age ranges in total) with equal distribution of males and females (7 males and 7 females). The sample size was determined on the basis of the Cohen’s d (Effect size calculators, University of Colorado) from the data reported in the previous study (standard difference between the estimated age and the chronological age) [8]. A sample size of at least 7 in each age/gender group will be required for a paired t-test with alpha = 0.05 and power = 0.8. The data that were used to test the applicability of French-Canadian and UK Caucasian reference datasets were re-used in the current study [9, 10].

Subjects with developmental delay and those with dental anomalies were excluded from the analysis. The DPT was scanned at a resolution of 300 dots per inch using a scanner (Canon, Canon Inc., Japan). The digitised DPT was viewed on a widescreen monitor (Philips 201E, Philips Electronics, Taiwan) at a standard magnification of 160% using Microsoft Office Picture Manager (Microsoft Corp, USA). A single trained and calibrated examiner (JJ) with inter- and intra-reliability kappa scores of 0.85 and 0.81 respectively, scored all of the radiographic images blinded to the gender and chronological age of the subject. All of the maxillary and mandibular permanent teeth on the left side were scored using the Demirjian classification of staging of dental development [6]. Each of the tooth developmental stages (TDS) has a designated letter of A to H indicating the stage of development from early crown formation through to closure of the root apex. Bilaterally missing teeth were not scored and when a single tooth was missing on the left side, the corresponding tooth on the right side was substituted. Chronological age was calculated from the patient’s birth date as recorded in the case notes. For the UK Caucasian study, the mean and standard error for each TDS were obtained from the southern Chinese reference dataset and the dental age was calculated using meta-analysis calculation (STATA, Stata Corp, Version 9.0). The UK Caucasian reference data were formulated from the dental development of children in London [8]. Similarly, the dental maturity scores from Demirjian’s French Canadian dataset were obtained and the corresponding ages were estimated by converting the overall maturity scores to an average dental age [6].

To estimate the age from the southern Chinese reference dataset, mean age corresponding to the stage of development for individual tooth was obtained from the southern Chinese RDS. This reference dataset was developed from 2306 children and young adults living in Hong Kong and has been tested for accuracy [11]. The chronological age and dental age calculated from the French-Canadian dataset, UK Caucasian dataset and the southern Chinese Reference datasets were exported to Excel (Microsoft Excel, Microsoft Corp, US) and the evaluation was conducted independently for males and females. Statistical significance was set at *p* < 0.05 and paired t-test was conducted between the chronological age (CA) and dental age (DA) obtained from the three different datasets using SPSS software (SPPS IBM Inc., Chicago, IL). To calculate the proportion of subjects within a specific time interval, the number (n) and percentage (%) of the test subjects within 3, 6, 9, 12, 18, 24 and 30 months of chronological and dental age (CA-DA) was analyzed using Chi-square tests with statistical significance set at *p* < 0.05 [13].

## Results

**Fig. 1:**
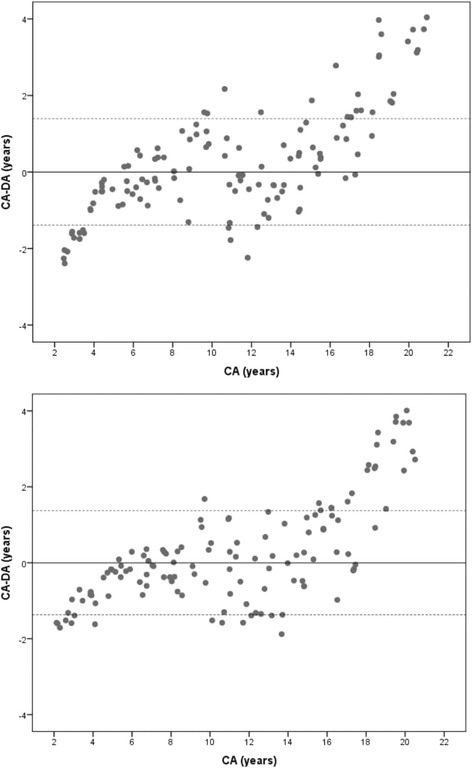
Bland & Altman plots showing the agreement between Chronological Age (CA) and Dental Age (DA) of males (above) and females (below) estimated from the UK Caucasian reference dataset

**Fig. 2:**
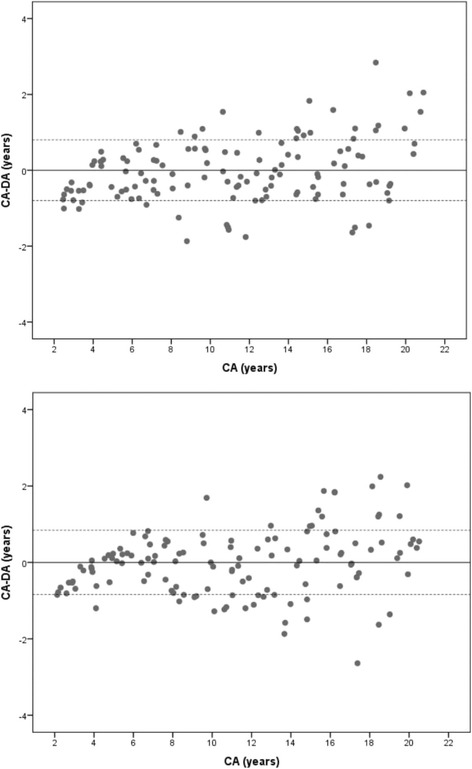
Bland & Altman plots showing the agreement between Chronological Age (CA) and Dental Age (DA) of males (above) and females (below) estimated from the southern Chinese reference dataset

**Fig. 3:**
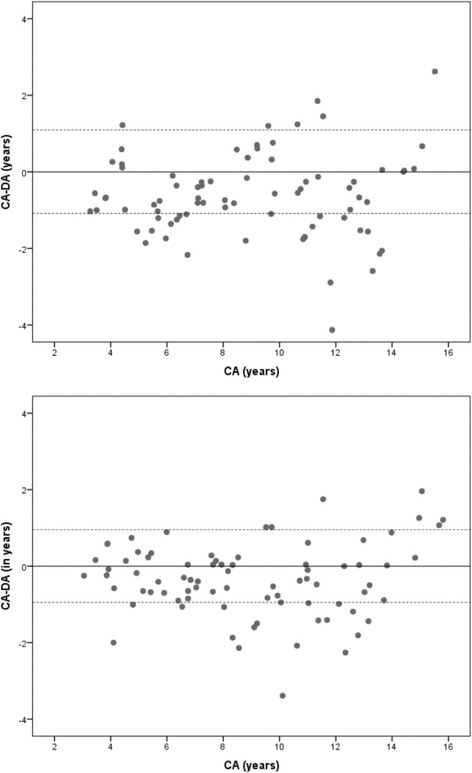
Bland & Altman plots showing the agreement between Chronological Age (CA) and Dental Age (DA) of males (above) and females (below) estimated from the Demirjian French Canadian dataset

The sample size was 266 (133 males and 133 females), but age estimation could be conducted only on 252 subjects for the UK Caucasian dataset, 157 subjects for the French-Canadian dataset and 254 subjects for the southern Chinese dataset (Table 1). This was due to variation in dental development patterns between different ethnic populations. The overall mean difference between the chronological age and dental age (CA-DA) calculated from Demirjian’s French-Canadian dataset was − 0.36 years for females and − 0.62 years for male (over estimates); the difference was 0.23 years for females and 0.25 years for males when estimated from the UK Caucasian dataset (under estimates). The southern Chinese RDS was able to accurately estimate the age at − 0.02 years for both females and males, see Table 1. Paired t-test conducted between the chronological age and the dental age estimated from the Demirjian’s dataset were statistically significantly different for both males and females (*p* < 0.05). Using the UK Caucasian dataset, the difference was statistically significant for males, but marginally non-significant for females (*p* = 0.056). For both genders, ages estimated from southern Chinese were not statistically significantly different (*p* > 0.05), see Table 1. Bland & Altman plots [14] were constructed from the chronological age and the difference of chronological age and dental age (CA-DA), see Figs. 1, 2, and 3.

**Table 1:** Differences between the Chronological Age (CA) and Dental Age (DA) in males and females (in years) using the UK Caucasian, French-Canadian and southern Chinese datasets

Reference Dataset	Males					Females				
	n	CA	DA	CA-DA	t-test	n	CA	DA	CA-DA	t-test
UK Caucasian	124	11.02	10.77	0.25	0.056	128	11.20	10.96	0.23	0.049*
French-Canadian	78	8.93	9.55	−0.62	0.001*	79	8.92	9.28	−0.36	0.001*
Southern Chinese	126	11.14	11.16	−0.02	0.786	128	11.20	11.22	−0.02	0.782

*statistically significant value, p < 0.05

The southern Chinese RDS estimated the age of 45% of the subjects to within ±6 months and 80% within ±12 months. The southern Chinese dataset was able to estimate the age of 90% of males accurately within a range of ±18 months compared to 77% and 72% from the French-Canadian and UK Caucasian datasets, respectively (Table 2). Similarly, 91% of females were estimated within a range of ±18 months using the southern Chinese dataset compared to 86% and 77% from the French-Canadian and UK Caucasian datasets, respectively (Table 3). Chi-square tests were conducted to evaluate the proportion within specific time interval from chronological age and dental age (CA-DA) estimated using the three datasets. This test demonstrated statistically significant differences at all of the time intervals for CA-DA in both genders, except for 3 and 6 months in females (*p* < 0.05).

**Table 2:** The number and proportion of males within specific time interval from chronological and dental age (CA-DA)

Time intervals	Datasets			Chi-square test
	French-Canadian	UK Caucasian	Southern Chinese	
	*n (%)*	*n (%)*	*n (%)*	*p*
3 months	10 (13%)	18 (15%)	24 (19%)	0.001*
6 months	21 (27%)	42 (34%)	57 (45%)	0.022*
9 months	34 (44%)	61 (49%)	86 (68%)	0.001*
12 months	44 (56%)	74 (60%)	101 (80%)	0.001*
18 months	60 (77%)	89 (72%)	114 (90%)	0.001*
24 months	71 (91%)	106 (85%)	124 (98%)	0.001*
30 months	74 (95%)	114 (92%)	126 (100%)	0.006*
Total	78 (100%)	124 (100%)	126 (100%)	–

*statistically significant value, p < 0.05

**Table 3:** The number and proportion of females within specific time interval from chronological and dental age (CA-DA)

Time intervals	Datasets			Chi-square test
	French-Canadian	UK Caucasian	Southern Chinese	
	***n (%)***	***n (%)***	***n (%)***	***p***
3 months	19 (24%)	27 (21%)	35 (27%)	0.505
6 months	31 (39%)	49 (38%)	58 (45%)	0.480
9 months	46 (58%)	59 (46%)	83 (65%)	0.009*
12 months	56 (71%)	75 (59%)	102 (80%)	0.001*
18 months	68 (86%)	99 (77%)	117 (91%)	0.007*
24 months	74 (94%)	113 (88%)	126 (98%)	0.005*
30 months	78 (99%)	116 (91%)	128 (100%)	0.001*
Total	79 (100%)	128 (100%)	128 (100%)	–

*statistically significant value, p < 0.05

## Discussion

Ethnic variations in dental maturity have been established and several investigators have expressed the need for ethnic specific standards for age estimation [15, 16]. This study has demonstrated that the southern Chinese reference data is the most accurate reference standard for estimating the age of children in Hong Kong. The accuracy can be attributed to the population specific reference standards; both the reference data and the study sample belong to the population group. The UK Caucasian and southern Chinese RDS were used to estimate the age of subjects in all of the age ranges. By contrast, only 60% of the subject’s ages were estimated using the Demirjian French-Canadian dataset. The maturation factors corresponding to the development of third molars were not included in the Demirjian analysis and hence it is impossible to perform age assessments for children above the age of 16 years [6]. The subjects included in this study belonged to southern Chinese ethnicity and this information was obtained from the data in the well documented hospital files. The subjects’ ethnicity was further confirmed from the names of the subjects that were specific to southern Chinese ancestry. The subjects whose parents were from southern China were alone included in the study but any ethnic admixtures exceeding two generations could not be clearly verified.

Age difference (CA-DA) calculated from the methods employed in the current study demonstrated a specific pattern of distribution. The UK Caucasian RDS underestimated the age of male and female children below 5 years of age by one year. A minor difference was observed in the 6- to 16-year age range. Overestimation of the age of about 2.40 years was seen in the young adults aged 17 years and older. The French-Canadian dataset was able to estimate the age of most of the children within a range of one year; however, overestimation of age was observed in the adolescents. The southern Chinese reference dataset consistently estimated the age of children within one year of difference. Nevertheless, some variations were observed in female adolescents. The major differences in the estimated age were found in males and females aged 18 years and older.

Both UK Caucasian and the French-Canadian datasets were unable to estimate the age of southern Chinese subjects accurately hence necessitating to develop population specific reference data. This was eventually developed from 2306 subjects aged 2 to 25 years and validated for accuracy in the same age group. The overall difference between CA and DA from the validation study was 0.05 years for males and 0.03 years for females [11]. The DPTs of subjects used in the earlier study were re-used in the current study for two reasons; firstly, to reconfirm the validity of the southern Chinese dataset and secondly, to identify differences in the estimated age using the same sample, but on three different datasets [9, 10]. In the current study, the difference between the CA and DA using southern Chinese dataset was − 0.02 years for both males and females and the difference was statistically insignificant proving that the dataset was more accurate than the UK Caucasian and French-Canadian datasets. This finding is consistent with the validation study [11, 17]. Although the total number of subjects included in the analysis was 266 with equal number of subjects in each age range (14 subjects in 19 age ranges), age could not be estimated for all subjects. The French-Canadian dataset does not include data for 3rd molars and hence could not be applied to estimate age of subjects over 16 years. Likewise, age of few subjects in the older age ranges could not be estimated due to difference in the timing of closure of root apices of third molars. For this reason, age could not be estimated for 12 subjects using the southern Chinese dataset and 14 subjects using the UK Caucasian dataset (Table 1).

The overall difference between CA and DA may be misleading at times since the extreme variations at certain age ranges may still produce result close to zero [10]. Hence, it is imperative to report the findings using a scatter plot to demonstrate variations in the age at different age ranges. However, when confronted in legal scenarios, a question commonly raised is “how accurate is the method?”. To answer this, we have reported frequency data to indicate the percentage accuracy of the estimated age at different time intervals starting from 3 months to 30 months. Using the southern Chinese dataset, the age of 65% of the subjects could be estimated within a range of 6 months and 80% within 12 months. The remaining 18% subjects could be estimated within 24 months interval, sparing just 2% beyond 2 years. The results are in conjunction with a study that tested the application of UK Caucasian dataset on the UK subjects with 42% of age estimates within a range of 6 months and 68% of age estimates within a range of one year [18].

Most of the methods of dental age calculation that have been reported in the literature were based on staging the degree of dental development, or the measurement of the apices of the roots. These data are then used to construct a reference standard for age assessments [19, 20]. The French-Canadian method utilizes correspondence analysis of the data to each stage of tooth development. The overall maturity score is then compared to the chart values to establish the dental age. By contrast, the UK Caucasian dataset contains data values as mean age or average AoA for each tooth development stage. This is a simple and effective method to estimate dental age compared to Demirjian’s system that requires multiple calculations. In addition to the average AoA, UK Caucasian dataset also contains the standard deviation for each stage of dental development which in turn permits weighted age calculations to be performed. In the validation study on southern Chinese subjects, meta-analysis computation was employed to calculate the dental age, which takes into account different weightings, i.e., number, mean age, standard deviation and standard error in the analysis. In this study, we have used the simple average of the mean ages corresponding to each TDS [10]. However, a study on multiple weighted average methods could provide us further information of the influence of weighting factors in age estimation for southern Chinese subjects.

## Conclusion

The population specific or ethnic specific reference dataset results in accurate estimates of age for southern Chinese children and young adults. These results were superior to those obtained using the UK Reference Dataset and the French Canadian Dataset on southern Chinese supporting the use of ethnic specific Reference Datasets for accurate age estimation.

## Abbreviations

AoA: Ages of attainment
CA: Chronological age
DA: Dental age
DAE: Dental age estimation
DPT: Dental panoramic tomograph
RDS: Reference data sets
UK: United Kingdom
